# Role of Oxidation-Dependent CaMKII Activation in the Genesis of Abnormal Action Potentials in Atrial Cardiomyocytes: A Simulation Study

**DOI:** 10.1155/2020/1597012

**Published:** 2020-06-20

**Authors:** Na Zhao, Qince Li, Haibo Sui, Henggui Zhang

**Affiliations:** ^1^School of Computer Science and Technology, Harbin Institute of Technology, Harbin 150000, China; ^2^Peng Cheng Laboratory, Shenzhen 518000, China; ^3^School of Physics and Astronomy, University of Manchester, Manchester M13 9PL, UK

## Abstract

Atrial fibrillation is a common cardiac arrhythmia with an increasing incidence rate. Particularly for the aging population, understanding the underlying mechanisms of atrial arrhythmia is important in designing clinical treatment. Recently, experiments have shown that atrial arrhythmia is associated with oxidative stress. In this study, an atrial cell model including oxidative-dependent Ca^2+^/calmodulin- (CaM-) dependent protein kinase II (CaMKII) activation was developed to explore the intrinsic mechanisms of atrial arrhythmia induced by oxidative stress. The simulation results showed that oxidative stress caused early afterdepolarizations (EADs) of action potentials by altering the dynamics of transmembrane currents and intracellular calcium cycling. Oxidative stress gradually elevated the concentration of calcium ions in the cytoplasm by enhancing the L-type Ca^2+^ current and sarcoplasmic reticulum (SR) calcium release. Owing to increased intracellular calcium concentration, the inward Na^+^/Ca^2+^ exchange current was elevated which slowed down the repolarization of the action potential. Thus, the action potential was prolonged and the L-type Ca^2+^ current was reactivated, resulting in the genesis of EAD. Furthermore, based on the atrial single-cell model, a two-dimensional (2D) ideal tissue model was developed to explore the effect of oxidative stress on the electrical excitation wave conduction in 2D tissue. Simulation results demonstrated that, under oxidative stress conditions, EAD hindered the conduction of electrical excitation and caused an unstable spiral wave, which could disrupt normal cardiac rhythm and cause atrial arrhythmia. This study showed the effects of excess reactive oxygen species on calcium cycling and action potential in atrial myocytes and provided insights regarding atrial arrhythmia induced by oxidative stress.

## 1. Introduction

Atrial fibrillation (AF) is the most common cardiac arrhythmia [1–3]. To design optimal treatment of AF, the mechanisms underlying AF need to be better understood. Both reactive oxygen species (ROS) and Ca^2+^/calmodulin- (CaM-) dependent protein kinase II (CaMKII) have been shown to be associated with the development of cardiac arrhythmias [4, 5]. The kinase CaMKII is ubiquitously expressed in the cardiomyocytes [4, 6]. It is involved in numerous cellular signaling cascades, such as phosphorylation of L-type Ca^2+^ channels [7–9], Na^+^ channels [10, 11], ryanodine receptors (RyRs) [12–18], and phospholamban (PLB) [12, 17, 19, 20]. Overexpression of CaMKII increases fractional sarcoplasmic reticulum (SR) Ca^2+^ release [13] and SR Ca^2+^ leakage [16], enhancing the activation of RyRs during both systole and diastole. As mentioned, it has been reported that CaMKII can also phosphorylate PLB [20]. In its unphosphorylated state, PLB acts as an endogenous inhibitor of sarco/endoplasmic reticulum Ca^2+^-ATPase (SERCA). Therefore, phosphorylation of PLB by CaMKII will enhance SERCA activity [21]. In addition to SR Ca^2+^ dynamics, many ion channels show CaMKII-dependent phosphorylation. Phosphorylation of L-type Ca^2+^ channels induced by CaMKII is associated with Ca^2+^-dependent Ca^2+^ current facilitation [22, 23]; CaMKII-dependent phosphorylation of Na^+^ channels may cause an increase in the late sodium current, which predisposes cardiomyocytes to arrhythmias [10, 11].

Conventionally, CaMKII is activated by Ca^2+^-bound calmodulin (Ca-CaM). Recently, a novel mechanism of CaMKII activation, which is ROS dependent, has been revealed [19, 24]. Previous studies have demonstrated that oxidative stress is closely associated with cardiac arrhythmias through alteration of the electrical activity and intracellular calcium dynamics of cardiac myocytes [25–27]. Oxidative stress is the main manifestation of cell metabolism disorders with excessive ROS. This oxidation-induced CaMKII activation is in relation to apoptosis [28], sinus node dysfunction [29], heart injury [30], and arrhythmias [31]. Although both ROS and CaMKII are associated with cardiac arrhythmias, the role of oxidative-dependent CaMKII activation in the development of atrial arrhythmias is not yet well understood.

In previous studies of cardiac arrhythmias, *in vivo* and *in vitro* experiments provide insights into mechanisms underlying arrhythmogenesis [5, 31]. However, these approaches have several limitations. For example, it is difficult to record the electrophysiological properties at different physical scales (from subcellular level to organ level) at the same time in one experiment. The electrophysiological properties recorded from different experiments, such as patch clamp and optical mapping, might be affected by gradual changes in cell or tissue properties. In addition to *in vivo* and *in vitro* experiments, a common method widely used in cardiac arrhythmia studies is *in silico* modeling. In this study, to simulate the effects of ROS-dependent CaMKII activation on regulations of intracellular Ca^2+^ and ionic currents, we first developed a Markov chain model of CaMKII, including both the autophosphorylation and oxidation pathways. Then, the effect of CaMKII on related proteins and ion channels was integrated to establish a computational model of the atrial cell to simulate changes in the electrophysiology under oxidative stress conditions, including ion currents, ion concentrations, calcium cycling, and transmembrane potential. Finally, the two-dimensional (2D) spiral wave was induced and the electrical excitation propagation was analyzed under normal and oxidative stress conditions.

## 2. Methods

### 2.1. CaMKII Model, including Both Autophosphorylation- and Oxidation-Dependent Activation

A six-state Markov chain model of CaMKII developed by our previous study of Zhang et al. [32] was incorporated into a human atrial model developed by Grandi et al. [33] to simulate autophosphorylation- and oxidation-dependent CaMKII activation.

The CaMKII monomer consists of three domains (an association domain, a regulatory domain, and a catalytic domain). When CaMCa4 (one calmodulin bound with four Ca^2+^) binds to the regulatory domain, the catalytic domain will be exposed, leading to CaMKII activation. In this state, CaMKII can be further autophosphorylated or oxidized, producing a long-lasting activation, even if CaMCa4 dissociates from CaMKII. Finally, CaMKII is fully deactivated by dephosphorylation with protein phosphatases, or reduction with methionine sulfoxide reductases (MsrA). Based on the four-state model of CaMKII developed by Chiba et al. [34], two oxidized states, with and without CaMCa4 binding with CaMKII, were added (see Figure S1 in the Supplementary Material for illustration), corresponding to the conformational change of oxidation-dependent CaMKII activation. Since there is no evidence to show that oxidation and autophosphorylation can occur at the same time, in this study, autophosphorylation and oxidation of CaMKII were treated as two independent processes [24]. The CaMKII autophosphorylation was based on the model developed by Chiba et al. [34]. The parameters of CaMKII oxidation were fitted to the experimental data recorded by Erickson et al. [24]. The detailed parameters of the CaMKII model are listed in Table S1 in the Supplementary Material.

### 2.2. Effects of CaMKII on Ion Channels

Previous studies showed that the ion channels influenced by CaMKII included the fast Na^+^ current (*I*_Na_) and the L-type Ca^2+^ current (*I*_CaL_) [7–11]. According to the method developed by O'Hara et al. [35], the current of each ion channel (*I*) was divided into two parts:

(1) $$\begin{array}{c} I=\left(1-\Phi \right)I_{\text{base}}+\Phi I_{\text{CaMK}}, \end{array}$$

where *I*_base_ is the part of ion channel current not affected by CaMKII, *I*_CaMK_ is the part of ion channel current affected by CaMKII, and *Φ* is the proportion affected by CaMKII using the following equations:

(2) $$\begin{array}{c} \Phi =\frac{{\text{CaMK}}_{\text{active}}}{{\text{CaMK}}_{\text{active}}+K_{\text{CaMK}}}, \end{array}$$

where CaMK_active_ is the fraction of CaMKII activation and *K*_CaMKII_ is the Michaelis constant with the same value in O'Hara et al. [35]. For *I*_Na_, the time constant of gate *j* was slowed down by 1.46-fold [32]. And for *I*_CaL_, experimental data showed that CaMKII activation produced an increased amplitude and a slowed inactivation of *I*_CaL_ [36]. Therefore, in this study, the time constant of gate *f* was slowed down by 1.5-fold and the part of *I*_CaL_ affected by CaMKII was increased by Δ*I*_CaL,CaMK_ shown in Equation (3).

(3) $$\begin{array}{c} {\Delta I}_{\text{CaL},\text{CaMK}}=\frac{{\text{CaMKII}}_{\text{active}}^{7}}{{\text{CaMKII}}_{\text{active}}^{7}+K_{\text{CaMK}}}. \end{array}$$

### 2.3. Effects of CaMKII on Ca^2+^ Cycling

Both RyR and PLB are primary regulatory proteins, controlling SR Ca^2+^ release and uptake; these are crucial processes, maintaining the balance of intracellular Ca^2+^. CaMKII activation-induced RyR phosphorylation can increase RyR opening probability and SR Ca^2+^ release. CaMKII activation-induced PLB phosphorylation can reduce SERCA inhibition, increasing SR Ca^2+^ uptake during diastole. The RyR and PLB models developed by Soltis and Saucerman [37] were used to simulate the effects of CaMKII activation on intracellular Ca^2+^ cycling. The rate constants controlling RyR opening (*k*_oSRCa_) and SR leakage (*k*_leak_) were modified (Equations (4) and (5)) under CaMKII activation to increase RyR opening in both systolic and diastolic conditions. Meanwhile, the half maximal saturation constant of SERCA (*K*_*mf*_) was modified (Equation (6)) to mimic the phenomenon of increased SERCA pump calcium sensitivity, which is induced by Thr17 phosphorylation of PLB under CaMKII activation.

(4) $$\begin{array}{c} k_{\text{oSRCa}}=\left(\frac{\left[{\text{RyR}}_{2815p}\right]}{\left[{\text{RyR}}_{\text{Tot}}\right]}\frac{20}{3}-\frac{1}{3}\right)\frac{k_{\text{oCa}}}{k_{\text{CaSR}}}, \end{array}$$

(5) $$\begin{array}{c} k_{\text{leak}}=2.5\frac{\left[{\text{RyR}}_{2815p}\right]}{\left[{\text{RyR}}_{\text{Tot}}\right]}+0.5, \end{array}$$

(6) $$\begin{array}{c} K_{mf}=K_{mf}\left(1-0.5\frac{\left[{\text{PLB}}_{T17p}\right]}{\left[{\text{PLB}}_{\text{Tot}}\right]}\right). \end{array}$$

### 2.4. Single Atrial Cell Model

The cell membrane of an atrial myocyte was mimicked as an electrical circuit. The AP in a human atrial cell was calculated using the following ordinary differential equation [33]:

(7) $$\begin{array}{c} C_{\text{m}}\frac{dV_{\text{m}}}{dt}=-I_{\text{ion}}+I_{\text{stim}}, \end{array}$$

where *C*_m_ is the cell capacitance, *V*_m_ is the transmembrane voltage, *t* is time, *I*_ion_ is the total transmembrane ionic current, and *I*_stim_ is the stimulus current. *I*_ion_ was calculated as

(8) $$\begin{array}{c} I_{\text{ion}}=I_{\text{Na}}+I_{\text{Nabk}}+I_{\text{to}}+I_{\text{Kur}}+I_{\text{Kr}}+I_{\text{Ks}}+I_{\text{K}1}+I_{\text{NaK}}+I_{\text{CaL}}+I_{\text{Cabk}}+I_{\text{pCa}}+I_{\text{NCX}}+I_{\text{ClCa}}+I_{\text{Clbk}}, \end{array}$$

where *I*_Na_ is the fast Na^+^ current, *I*_Nabk_ is the background Na^+^ current, *I*_to_ is the transient outward K^+^ current, *I*_Kur_ is the ultrarapid delayed rectifier K^+^ current, *I*_Kr_ is the rapid activating K^+^ current, *I*_Ks_ is the slowly activating K^+^ current, *I*_K1_ is the inward rectifier K^+^ current, *I*_NaK_ is the Na^+^/K^+^ pump current, *I*_CaL_ is the L-type Ca^2+^ current, *I*_Cabk_ is the background Ca^2+^ current, *I*_pCa_ is the sarcolemmal Ca^2+^ pump current, *I*_NCX_ is the Na^+^/Ca^2+^ exchange current, *I*_ClCa_ is the Ca^2+^-activated Cl^−^ current, and *I*_Clbk_ is the background Cl^−^ current.

In this study, 0.5, 1, and 2 Hz stimulation frequencies were used to investigate the frequency dependency of CaMKII activation. Action potentials of atrial cells were produced by applying a stimulus current with an amplitude of −12.5 pA/pF and a duration of 5 ms. The time step used in the simulation was 0.1 ms using the CVODE solver of the SUite of Nonlinear and DIfferential/ALgebraic equation Solvers (SUNDIALS) to solve initial value problems for ordinary differential equation systems. These simulation results were consistent with the ones using forward Euler with a time step of 0.005 ms. Under the control condition, the value of H_2_O_2_ in the CaMKII model was 0 *μ*M, and the one was 200 *μ*M under the oxidative stress condition. To ensure that the model reached a quasistable steady state, the simulations were carried out for more than 50 s under the control condition and 150 s under the oxidative stress condition.

### 2.5. Excitation Wave Conduction in 2D Tissue

In this study, a monodomain model was used for 2D simulations in an ideal square tissue. The excitation wave propagation was simulated using the following equation:

(9) $$\begin{array}{c} \frac{\partial V_{\text{m}}}{\partial t}=-\frac{I_{\text{ion}}}{C_{\text{m}}}+\nabla \left(D\nabla V_{\text{m}}\right), \end{array}$$

where **D** is the diffusion tensor describing the conductivity of the tissue and ∇ is the spatial gradient operator.

**Figure 1:**
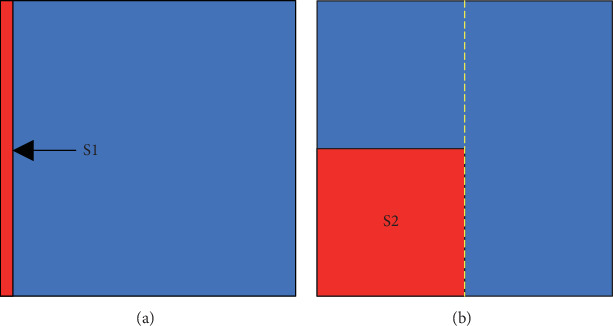
The S1–S2 protocol used in 2D simulations. The red bar in (a) and the red square in (b) represent the regions where S1 and S2 stimuli were applied.

In 2D simulations, the 2D ideal isotropic tissue was constructed as a sheet of 1000 × 1000 nodes to investigate the stability of the spiral waves, as used in [38]. The time step Δ*t* was set at 0.005 ms, and the space step Δ*x* was 0.1 mm along both direction axes, as used in [39]. The S1–S2 stimulation protocol was used to produce spiral waves. An S1 stimulus was applied to the five column nodes on the left side to induce a plane wave (Figure 1(a)). After the nodes in the middle line (the dashed line in Figure 1(b)) reached the end point of the refractory period, an S2 stimulus was applied in the left lower quadrant to induce a spiral wave (Figure 1(b)).

The conduction velocity (CV) was measured using the following equation:

(10) $$\begin{array}{c} \text{CV}=\frac{x_{2}-x_{1}}{t_{2}-t_{1}}, \end{array}$$

where *x*_1_ and *x*_2_ are the positions and *t*_1_ and *t*_2_ are the times when the excitation reaches the two recording points. In 2D simulation, the tissue was isotropic and the diffusion tensor (*D*) along both direction axes was set to 0.029 mm^2^/ms to produce a CV of 69 cm/s in control conditions, which was consistent with the CV (70 cm/s) previously used for human atrial tissue simulation [40–42].

## 3. Results

### 3.1. Electrophysiological Properties of a Single Atrial Cell under Control Conditions

**Figure 2:**
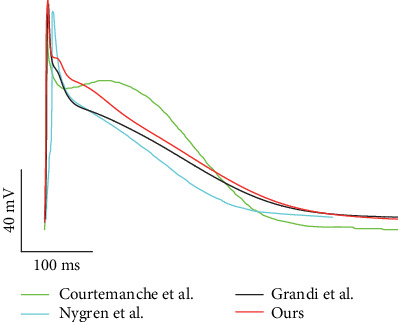
Action potential traces produced by our simulation and previous mathematical models and experiments at 1 Hz. Traces labeled “Courtemanche et al.,” “Nygren et al.,” and “Grandi et al.” were reproduced from mathematical models [33, 43, 44].

The action potential (AP) in a single-cell model was generated by applying a series of 1 Hz stimuli. The time trace of the simulated AP is presented in Figure 2, along with other AP traces from previous mathematical models [33, 43, 44]. The resting membrane potential (RMP), maximum upstroke velocity (*dV*/*dt*_max_), action potential amplitude (APA), and action potential duration at 90% repolarization (APD_90_) were measured as −75.2 mV, 160 mV/ms, 110 mV, and 312 ms, respectively. These parameters were consistent with previous models developed by Grandi et al. [33], Courtemanche et al. [43], and Nygren et al. [44], and APD_90_ was within the scope of previously reported experimental data [45–48], as listed in Table 1.

**Figure 3:**
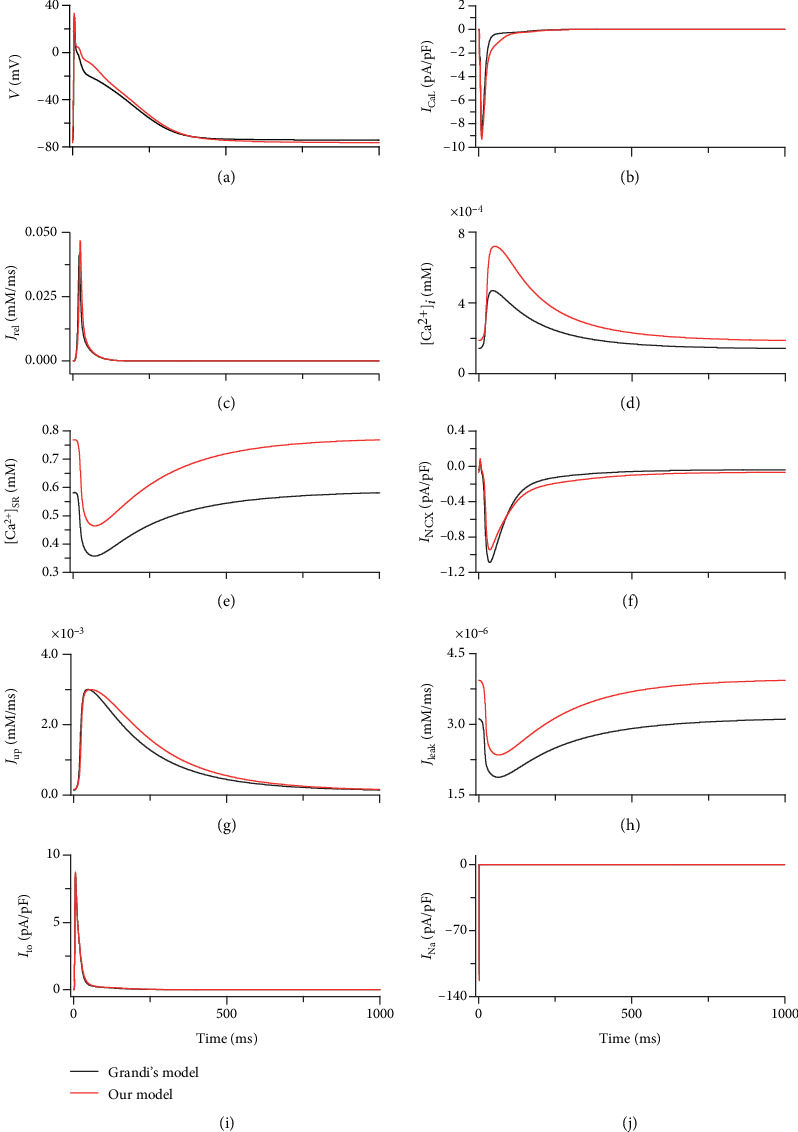
Traces of AP, ionic currents, and calcium cycling produced by our model (red) and the model of Grandi et al. [33] (black). (a) AP. (b) *I*_CaL_. (c) SR Ca^2+^ release (*J*_rel_). (d) intracellular Ca^2+^ concentration ([Ca^2+^]_*i*_). (e) SR Ca^2+^ concentration ([Ca^2+^]_SR_). (f) *I*_NCX_. (g) SR Ca^2+^ reuptake (*J*_up_). (h) SR Ca^2+^ leakage (*J*_leak_). (i) *I*_to_. (j) *I*_Na_.

To further validate the model, the ionic currents and intracellular calcium cycling process were investigated and compared with those from the model of Grandi et al. [33] at 1 Hz, as shown in Figure 3. These results demonstrated an elevation in the repolarization phase of the AP and, therefore, a longer action potential duration (APD) in our model (Figure 3(a)). This was attributed to the change in *I*_CaL_. In our model, *I*_CaL_ showed a larger amplitude and a slowed inactivation (Figure 3(b)), which was consistent with the effect of CaMKII activation on *I*_CaL_ [36]. In addition to the AP, the variation in *I*_CaL_ altered intracellular calcium regulation, as *I*_CaL_ was the main influx of intracellular Ca^2+^. The increase in *I*_CaL_ caused a larger Ca^2+^ influx, leading to a larger SR Ca^2+^ release (Figure 3(c)) and thus a larger intracellular Ca^2+^ concentration (Figure 3(d)). The accumulation of intracellular Ca^2+^ finally caused an elevation in the concentration of SR Ca^2+^ (Figure 3(e)). Interestingly, the peak inward *I*_NCX_ in our model declined even when the intracellular Ca^2+^ concentration increased (Figure 3(f)). This may be explained by the fact that the elevation of the membrane potential during the repolarization phase suppressed the inward Na^+^ flux and decreased *I*_NCX_.

To justify the increase of intracellular Ca^2+^ concentration in our model, the Ca^2+^ concentration was compared with previous studies. The calcium transient amplitude in our model was ~0.53 *μ*M, which was consistent with the studies of Courtemanche et al. and Colman et al. (0.45–0.55 *μ*M) [43, 49], although the Ca^2+^ transient amplitude in the study of Nygren et al. was reported to be even larger (~1.2 *μ*M) [44].

**Figure 4:**
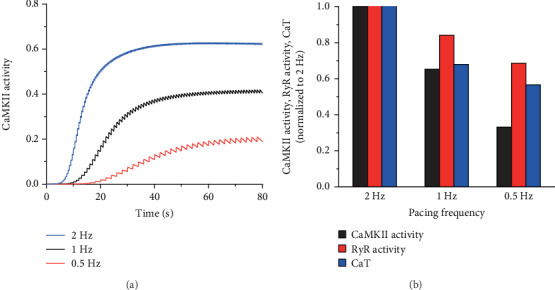
Frequency-dependent activation of CaMKII. (a) CaMKII activation curves at 0.5, 1, and 2 Hz in 80 s. (b) Normalized CaMKII activation, RyR calcium release (RyR Activity), and intracellular Ca^2+^ transient (CaT) to 2 Hz at 0.5, 1, and 2 Hz.

The variations in *I*_CaL_ and intracellular calcium cycling were caused by adding the effect of CaMKII activation in our model. Previous studies demonstrated that CaMKII activation was significantly influenced by pacing frequencies. In our simulation, CaMKII activation and intracellular Ca^2+^ dynamics were investigated at 0.5, 1, and 2 Hz.  Figure 4(a)shows that the fraction of CaMKII activation increased with increasing pacing rate. Meanwhile, the time required for CaMKII activation was also decreased, indicating a faster activation at a higher pacing rate (Figure 4(a)). The frequency-dependent activation of CaMKII was mainly due to the intracellular Ca^2+^ concentration. With increasing pacing rate, both RyR Ca^2+^ release and intracellular Ca^2+^ concentration increased (Figure 4(b)), causing an increase in CaMCa4 and, therefore, augmenting CaMKII activation.

### 3.2. Electrophysiological Properties of a Single Atrial Cell under Oxidative Stress Conditions

**Figure 5:**
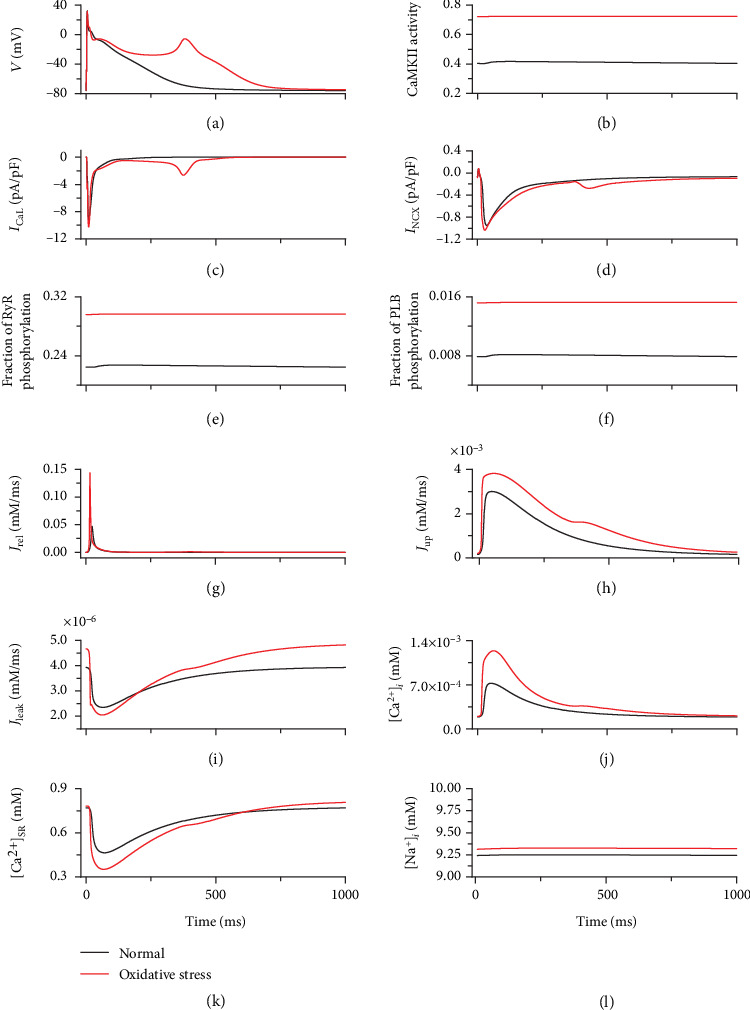
Traces of AP, ionic currents, and calcium cycling under normal (black) and oxidative stress (red) conditions. (a) AP. (b) CaMKII activity. (c) *I*_CaL_. (d) *I*_NCX_. (e) Fraction of RyR phosphorylation. (f) Fraction of PLB phosphorylation. (g) *J*_rel_. (h) *J*_up_. (i) *J*_leak_. (j) [Ca^2+^]_*i*_. (k) [Ca^2+^]_SR_. (l) Intracellular Na^+^ concentration ([Na^+^]_*i*_).

The simulation results showed that an EAD was induced in the AP under conditions of oxidative stress at a pacing rate of 1 Hz (Figure 5(a)). In this case, the elevated ROS concentration (0.2 mM) induced a significant increase in CaMKII activation (Figure 5(b)). ROS-enhanced CaMKII activation further enlarged *I*_CaL_, *I*_NCX_, fraction of RyR phosphorylation, and fraction of PLB phosphorylation (Figures 5(c)–5(f)). Under oxidative stress conditions, the intracellular Ca^2+^ dynamics were remarkably changed. Enhanced RyR and PLB phosphorylation caused significant increases in *J*_rel_, *J*_up_, and *J*_leak_ (Figures 5(g)–5(i)), which further induced a dramatic increase in intracellular Ca^2+^ concentration (Figure 5(j)) and a decrease in minimum SR Ca^2+^ (Figure 5(k)). However, there was no obvious change in the intracellular Na^+^ concentration (Figure 5(l)).

**Figure 6:**
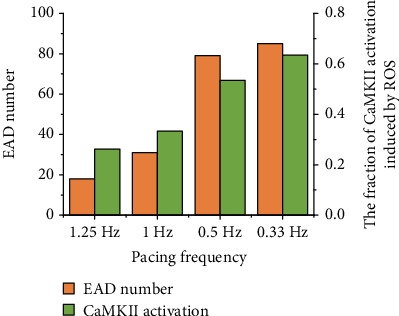
Number of EAD occurring in the same duration (orange) and the fraction of CaMKII activation induced by ROS (green) at different pacing rates under oxidative stress conditions.

As CaMKII activation showed frequency-dependent behavior, ROS-induced EADs are rate dependent as well. Under oxidative stress conditions, the occurrence of EADs in 50 s became more prominent with decreasing pacing rate, as shown in Figure 6. Under control conditions, the CaMKII activation (without ROS-induced activation) gradually decreased with decreasing pacing rate (Figure 4(b)). This meant that more CaMKII could be activated by ROS under oxidative conditions at low pacing rates. Consequently, the CaMKII activation induced by ROS gradually increased with decreasing pacing rate (green bars in Figure 6). Therefore, under oxidative stress conditions, more EADs were induced at low pacing rates.

### 3.3. Mechanisms Underlying the Genesis of EAD under Oxidative Stress Conditions

**Figure 7:**
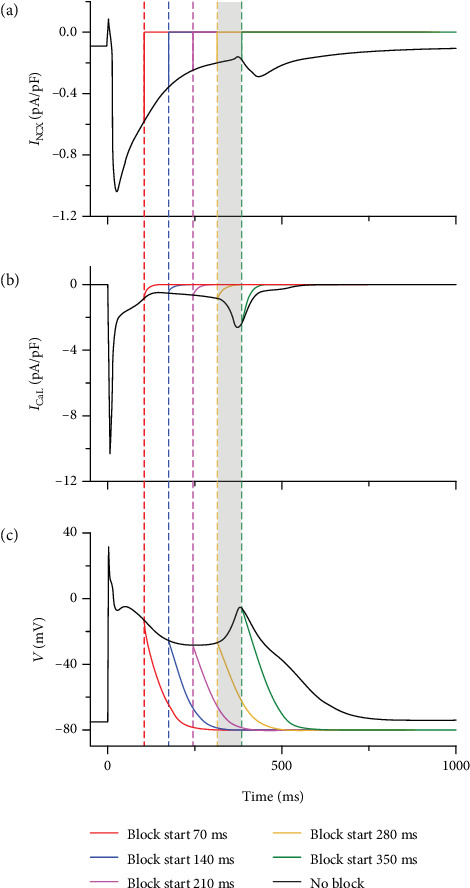
Effects of blocking *I*_NCX_ at different times on the genesis of EAD under oxidative stress condition: (a) *I*_NCX_. (b) *I*_CaL_. (c) AP. The gray box represents the time interval during which EAD can occur while blocking *I*_NCX_.

In the case of EAD, *I*_CaL_ and *I*_NCX_ were the main currents significantly influenced by oxidative stress. Blocking *I*_CaL_ at the point of *I*_CaL_ reactivation abolished EAD (data not shown), implying that *I*_CaL_ was a main factor contributing to the genesis of EAD. However, blocking *I*_NCX_ did not guarantee elimination of EAD.  Figure 7 indicated the effects of blocking *I*_NCX_ at different times from the point of *I*_CaL_ reactivation (70 ms) to the time of the peak of EAD (350 ms). With the delay in blocking *I*_NCX_ (Figure 7(a)), the reactivation of *I*_CaL_ became more prominent (Figure 7(b)) and the APD gradually increased (Figure 7(c)). When blocking *I*_NCX_ in the interval 70–210 ms, the reactivation of *I*_CaL_ did not induce AP depolarization and therefore abolished EAD. On blocking *I*_NCX_ during the interval 210–280 ms, the enlarged reactivation of *I*_CaL_ started to transfer AP repolarization to depolarization. However, in this time interval, AP depolarization was not obvious and no EAD was induced. When blocking *I*_NCX_ during the interval 280–350 ms, the reactivation of *I*_CaL_ was able to induce AP depolarization. Consequently, in this time interval, blocking *I*_NCX_ could not eliminate EAD (Figure 7(c)). Therefore, *I*_NCX_ played an important role in *I*_CaL_ reactivation and thus in triggering EAD.

**Figure 8:**
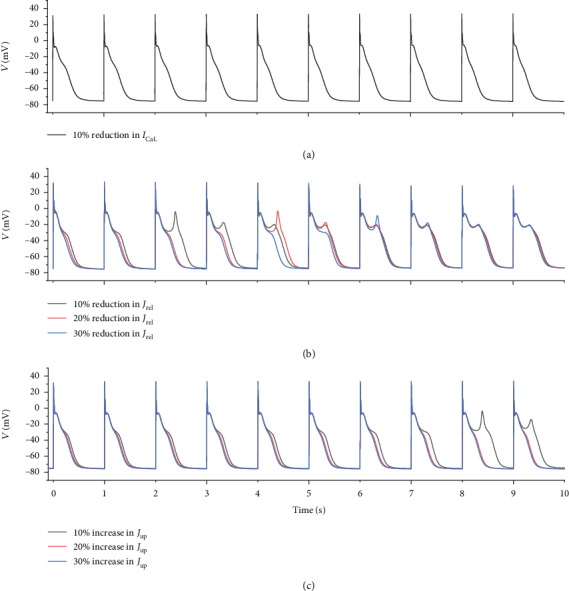
Effect of (a) reducing *I*_CaL_ by 10%, (b) reducing *J*_rel_ by 10%, 20%, and 30%, and (c) increasing *J*_up_ by 10%, 20%, and 30% on the genesis of EAD under oxidative stress conditions.

In our model, *I*_NCX_ was not directly regulated by ROS. As the main efflux of intracellular Ca^2+^, *I*_NCX_ was regulated by intracellular Ca^2+^ concentration; Ca^2+^ cycling was another important process affected by ROS-induced CaMKII activation. Three factors associated with intracellular cycling in our model were directly regulated by ROS-induced CaMKII activation, namely, *I*_CaL_, *J*_rel_, and *J*_up_. Under oxidative stress conditions, intracellular Ca^2+^ concentration significantly increased (Figure 5(j)). To investigate the role of [Ca^2+^]_*i*_ regulation in the genesis of EAD, decreased *I*_CaL_, reduced *J*_rel_, and enhanced *J*_up_ were independently applied to reduce the [Ca^2+^]_*i*_ under oxidative stress conditions, as shown in Figure 8. Figure 8(a) shows that decreasing *I*_CaL_ by 10% abolished EAD. This was due not only to [Ca^2+^]_*i*_ decline but also to a decrease in *I*_CaL_ reactivation. Reducing SR Ca^2+^ release postponed the occurrence of EAD but did not successfully eliminate EAD (Figure 8(b)). Although a reduction in SR Ca^2+^ release temporarily reduced [Ca^2+^]_*i*_, it caused Ca^2+^ accumulation in SR, which finally induced [Ca^2+^]_*i*_ elevation (data not shown) and EAD. Increasing SR Ca^2+^ uptake by 10% had a similar effect to that of reducing SR calcium release (Figure 8(c)). Nonetheless, further increasing *J*_up_ by 20%–30% abolished EAD. In these two cases, the SR Ca^2+^ uptake and release reached a new balance and maintained [Ca^2+^]_*i*_ and [Ca^2+^]_SR_ at a steady state (data not shown).

### 3.4. Effect of Oxidative Stress on Excitation Wave Propagation in 2D Tissue

**Figure 9:**
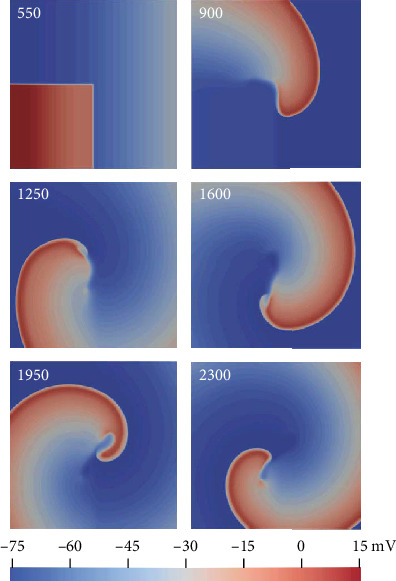
Spiral wave induced under control conditions using the S1–S2 protocol. The number in each panel represents the recording time.

**Figure 10:**
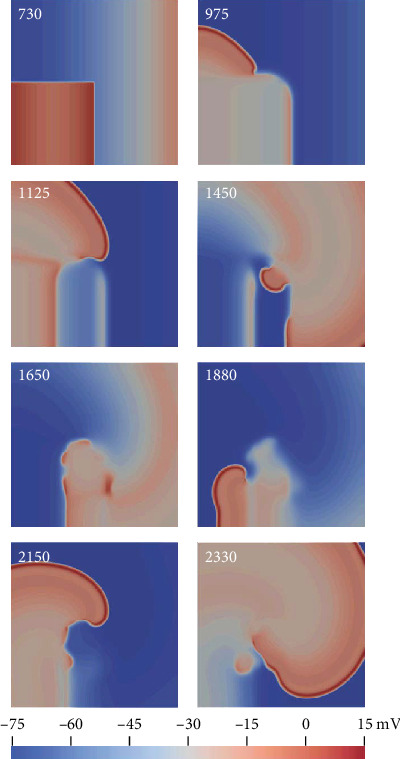
Spiral wave induced under oxidative stress conditions using the S1–S2 protocol. The number in each panel represents the recording time.

The excitation wave propagation was investigated in 2D ideal tissue under control and oxidative stress conditions. Spiral waves were induced using the S1–S2 protocol. Under control conditions with the S1–S2 interval of 550 ms, a stationary spiral wave was produced, with its tip anchored in the central region of the square tissue, as shown in Figure 9. Under oxidative stress conditions, the ROS concentration was set to 0.2 mM for all nodes in the tissue. In this case with the S1–S2 interval of 730 ms, a nonstationary spiral wave was induced. As the EAD was induced under this condition, the region to which the S2 stimulus was applied showed a prolonged depolarization (Figure 10, 1125 ms) and a second wavefront was induced at 1450 ms, as shown in Figure 10. When the wavefronts reached the region to which the S2 stimulus was applied (Figure 10, 1650 ms), the nodes were not fully repolarized, owing to the prolonged APD induced by EAD. Therefore, the excitation wave propagation was suppressed (Figure 10, 1650 ms). Finally, the tip of the spiral wave wandered across the tissue and produced a nonstationary spiral wave. When oxidative stress only occurred in the right third of the tissue, the spatial heterogeneity further aggravated the instability of the nonstationary spiral wave and gave rise to a breakup of the spiral wave (data not shown).

## 4. Discussion

In this study, the effects of ROS-dependent CaMKII activation on regulations of intracellular Ca^2+^ and ionic currents were investigated *in silico* using the updated human atrial cell model including the CaMKII model with autophosphorylation- and oxidation-dependent activation at the single-cell level and 2D tissue model. Our major findings follow: (i) A new Markov chain model of CaMKII, including both the autophosphorylation and oxidation pathways, was developed, and the effects of CaMKII on ion channels and Ca^2+^ cycling were incorporated into the computational model of the atrial myocyte. (ii) The mechanisms of oxidative stress-induced EADs in atrial cells were thoroughly examined which are helpful to further understand or investigate the mechanisms underlying oxidative stress-induced AF. The simulation results at the single-cell level illustrated that oxidative stress resulted in EADs of the AP at the normal pacing rate of 1 Hz. It was contributed by reactivation of *I*_CaL_ and intracellular Ca^2+^ elevation induced by CaMKII activation under oxidative stress conditions. (iii) The 2D simulations provide insights into reentry in atrial tissue under the oxidative stress condition, which plays significant roles in AF mechanisms. In 2D simulation, oxidative stress aggravated the instability of the excitation wave and gave rise to a nonstationary spiral wave, owing to the EAD induced by oxidative stress hindering the electrical conduction.

### 4.1. Model Development of ROS-Dependent CaMKII Activation

Previous experimental studies have revealed that both ROS and CaMKII are associated with the development of atrial arrhythmia [4, 5]. In addition, it has recently been reported that CaMKII can keep persistent activity by oxidation [24]. However, the role of ROS-dependent CaMKII activation in the genesis of atrial fibrillation is not yet well understood. In this study, we developed a computational model of human atrial cell including both ROS-dependent and autophosphorylation-dependent CaMKII activation to investigate effects of CaMKII activation on atrial electrophysiology under oxidative stress condition. In this CaMKII model, a pathway of ROS-dependent CaMKII activation was introduced which was different from previous CaMKII models only including the autophosphorylation-dependent CaMKII activation [34, 35, 50]. Christensen et al. [39] also developed a model of CaMKII activity including oxidation and autophosphorylation activation pathways. However, different from their model, the oxidation and autophosphorylation activation pathways in our six-state CaMKII are mutual independence, implying that there is no such a state with both oxidation and autophosphorylation activation simultaneously. This fact is consistent with the experimental observations reported in [24].

The effect of CaMKII on *I*_Na_, *I*_CaL_, RyR, and PLB was integrated into the human atrial myocyte model, producing action potential characteristics (such as RMP, *dV*/*dt*_max_, APA, APD_90_, and the calcium transient amplitude) consistent with previous models and experimental data of human atrial myocytes [33, 43–49] (Figure 2 and Table 1).

### 4.2. Mechanisms Underlying the Genesis of EAD under Oxidative Stress Conditions

The generation of EADs associated with ectopic (triggered) activity can be contributed by CaMKII activation [51–55]. Animal experiments have shown that oxidative stress can promote EADs in the ventricle of guinea pigs and rabbits [56]. Therefore, this study explored the intrinsic mechanisms of EADs associated with atrial arrhythmia induced by oxidative stress, using the developed CaMKII model.

First, simulation results demonstrated that the characteristics of AP and calcium cycling generated by our model were consistent with previous computational and experimental studies [43–49]. After model validation, the role of oxidation-dependent CaMKII activation on the AP was investigated in a single atrial cell model. The simulation results demonstrated that, under oxidative stress conditions, increasing ROS concentrations in the cytoplasm enhanced CaMKII activation and consequently augmented *I*_CaL_, RyR phosphorylation, and PLB phosphorylation (Figures 5(c), 5(e), and (f)), inducing an enhanced Ca^2+^ influx, a larger SR Ca^2+^ release, and a promoted SR Ca^2+^ uptake (Figures 5(g) and 5(h)). These effects generated a remarkable elevation in intracellular Ca^2+^ concentration and therefore enlarged *I*_NCX_ via promoted Ca^2+^ extrusion (Figure 5(d)). *I*_NCX_ augmentation provided a depolarization component to counterbalance the repolarization reserve and prolong the APD. The elevated and prolonged AP in phase 3 gradually caused reactivation of *I*_CaL_ (Figure 5(c)), which ultimately induced action potential depolarization and produced EAD (Figure 5(a)), consistent with previous studies [57–59].

In this process, as *I*_CaL_ was the direct depolarization current inducing EAD, blocking *I*_CaL_ completely abolished EAD. However, blocking *I*_NCX_ at different time points had different effects on EAD. If *I*_NCX_ was blocked before *I*_CaL_ reactivation could induce AP depolarization, EAD was eliminated (Figure 7). This phenomenon suggested that *I*_NCX_ augmentation acted as a trigger of *I*_CaL_ reactivation. EAD can be induced only when the trigger is large enough. Under oxidative stress condition, ROS elevation did not affect *I*_NCX_ directly and the increase of [Ca^2+^]_*i*_ primarily accounted for *I*_NCX_ augmentation. Therefore, the effects of ROS-induced CaMKII activation on intracellular calcium cycling also played a crucial role in the genesis of EAD.

To examine the role of ROS-induced [Ca^2+^]_*i*_ elevation in the genesis of EAD, Ca^2+^ influx and SR Ca^2+^ release and uptake were modified to reduce ROS-induced [Ca^2+^]_*i*_ elevation. First, *I*_CaL_ was reduced by 10% to inhibit Ca^2+^ influx, causing a decline of [Ca^2+^]_*i*_. Together with the decreased reactivation of *I*_CaL_, the [Ca^2+^]_*i*_ decline caused by reducing *I*_CaL_ abolished EAD (Figure 8(a)). Second, partial inhibition of SR Ca^2+^ release (by 10%–30%) temporarily reduced [Ca^2+^]_*i*_. However, inhibition of SR Ca^2+^ release gradually caused SR Ca^2+^ accumulation and ultimately gave rise to further [Ca^2+^]_*i*_ elevation, which accounted for the fact that partial inhibition of SR Ca^2+^ release could only postpone the occurrence of EAD but could not abolish EAD (Figure 8(b)). Increasing SR Ca^2+^ uptake by 10% had a similar effect to partial inhibition of SR Ca^2+^ release. Interestingly, on further increasing SR Ca^2+^ uptake by 20% and 30%, [Ca^2+^]_*i*_ declined and EAD was abolished. This phenomenon might be attributed to a new balance of increased SR Ca^2+^ uptake and ROS-induced [Ca^2+^]_*i*_ release (Figure 8(c)). Therefore, the dynamic balance between SR Ca^2+^ release and uptake is the key factor in maintaining [Ca^2+^]_*i*_ at a steady state.

### 4.3. Effect of Rate-Dependent CaMKII Activation on EAD under Oxidative Stress Conditions

Under control conditions, CaMKII activation gradually decreased with a decrease in the pacing rate, as Ca^2+^ concentration gradually decreased at low pacing rates. However, under oxidative stress conditions, once CaMKII was activated, the high ROS concentration tended to maintain CaMKII activation. Therefore, under oxidative stress conditions, fractions of CaMKII activation at different pacing rates were similar and maintained at a high level (about 75%). This indicated that, at low pacing rates, ROS-induced CaMKII activation played a more important role. At low pacing rates, intracellular Ca^2+^ concentration could be dramatically increased by ROS-induced CaMKII activation and therefore could induce more EADs (Figure 6). Although the heart rate is not so slow such as 0.5 Hz and 0.33 Hz under normal conditions, it may be such slow under some pathological conditions, such as bradycardia [60]. The purpose of exploring up to such slow pacing frequencies is to investigate whether the occurrence of EAD is linear with the decrease of pacing frequency or not. The results showed that the occurrence of EAD was more dependent on ROS-induced CaMKII activation rather than the pacing frequencies.

### 4.4. Effect of Oxidative Stress on Excitation Wave Propagation in 2D Tissue

The spiral wave is a well-known approach to understanding reentry in cardiac tissue, which plays significant roles in AF mechanisms [61]. This study investigated the stability of the spiral wave in 2D ideal tissue under control and oxidative stress conditions. In tissue with a uniform distribution of oxidative stress (Figure 10), the region to which the S2 stimulus was applied had a prolonged depolarization phase, owing to the ROS-induced EAD, forming a spiral wave barrier. In addition, the ROS-induced EAD produced a second excitation wavefront during wave propagation. These effects finally produced a nonstationary spiral wave. In the case of nonuniform distribution of oxidative stress, a breakup of the spiral wave was induced, which might disrupt normal cardiac rhythm and cause atrial arrhythmia [62–64].

### 4.5. Model Limitations and Future Directions

The present model was based on models of Grandi et al. [33] and O'Hara et al. [35] and thus inherited the same limitations of both models. The main limitation of the current model, which will be addressed in the future, is that the ROS concentration is constant. In future versions, pathways of ROS production and scavenging should be added. Another issue for model development is to expand the model into real 2D heart tissue and 3D organ models, to explore electrical wave propagation induced by abnormal action potentials, and electrocardiography in real heart geometry.

Although the S1–S2 interval in 2D simulation to produce the spiral wave was dependent on the tissue size, it is reasonable to investigate the difference in the stability of the spiral wave using the same tissue size under control and oxidative stress conditions. Further work is required to measure the range of S1–S2 intervals for producing the spiral wave (vulnerable window) varying from control to oxidative stress conditions to further determine the proarrhythmic effect of oxidative stress.

## 5. Conclusions

In this study, we have investigated the role of oxidation-dependent CaMKII activation in the genesis of abnormal action potentials in atria. It was shown that, at the atrial cell level, oxidation-dependent CaMKII activation-induced *I*_CaL_ reactivation and [Ca^2+^]_*i*_ elevation contributed to EAD generation. Moreover, at the 2D tissue level, oxidative stress-induced EAD contributed to the instability of excitation waves, facilitating atrial arrhythmia. This study investigated the role of ROS-dependent CaMKII activation in the development of atrial arrhythmias, shedding light on the genesis of atrial arrhythmias under oxidative stress conditions.

## Figures and Tables

**Table 1:** Comparison of action potential characteristics.

Model and experimental data	RMP (mV)	*dV*/*dt*_max_ (mV/ms)	APA (mV)	APD_90_ (ms)
Courtemanche et al. [43]	−79.5	216	106	297
Nygren et al. [44]	−76.4	116	103.8	245
Grandi et al. [33]	−74.5	135	105	294
Our model	−75.2	160	110	312
Dawodu et al. [45]	—	—	—	361 ± 71
Katoh et al. [46]	—	—	—	255 ± 39
Bosch et al. [47]	—	—	—	255 ± 45
Kim et al. [48]	—	—	—	258 ± 25

## Data Availability

The data used to support the findings of this study are available from the corresponding author upon request.

## Supplementary Materials

Supplementary Materials Figure S1: The CaMKII model. B, CaMKII-CaMCa4 (bound state); BO, CaMKIIOx-CaMCa4 (oxidized and bound state); BP, CaMKIIP-CaMCa4 (phosphorylated and bound state); I, CaMKII (inactive state); O, CaMKIIOx (oxidized state); P, CaMKIIP (phosphorylated state). Table S1: Parameters of CaMKII model
