# Supplementary material for: Role of Oxidation-Dependent CaMKII Activation in the Genesis of Abnormal Action Potentials in Atrial Cardiomyocytes: A Simulation Study
Source: Biomed Res Int. 2020 Jun 20;2020:1597012. doi: 10.1155/2020/1597012 (PMC7327560; doi:10.1155/2020/1597012)
Supplement: Supplementary Materials — Figure S1: The CaMKII model. B, CaMKII-CaMCa4 (bound state); BO, CaMKIIOx-CaMCa4 (oxidized and bound state); BP, CaMKIIP-CaMCa4 (phosphorylated and bound state); I, CaMKII (inactive state); O, CaMKIIOx (oxidized state); P, CaMKIIP (phosphorylated state). Table S1: Parameters of CaMKII model [file 1597012.f1.docx]

Supplementary materials for methods

**S1. CaMKII model**


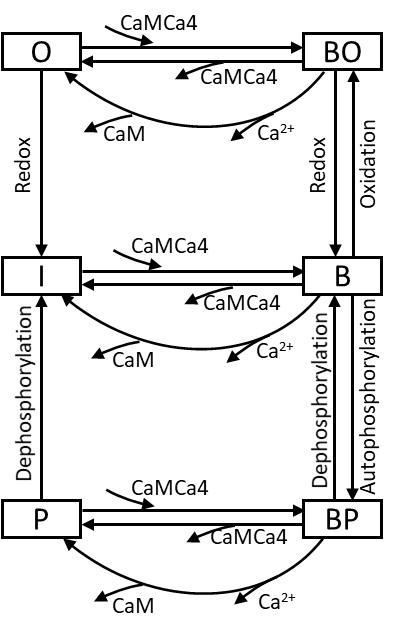


Figure S1: The CaMKII model. B, CaMKII-CaMCa4 (bound state); BO, CaMKIIOx-CaMCa4 (oxidized and bound state); BP, CaMKIIP-CaMCa4 (phosphorylated and bound state); I, CaMKII (inactive state); O, CaMKIIOx (oxidized state); P, CaMKIIP (phosphorylated state).

Table S1: Parameters of CaMKII model.

| Parameter | Definition | Value |
| --- | --- | --- |
| *k_*asso | Transition rate from I to B | 2.1 mM^−1^ ms^−1^ |
| *k_*disso | Disassociation rate of CaMCa4 from B (BO) | 0.7 × 10^−4^ ms^−1^ |
| *k_*dissoCa | Disassociation rate of Ca^2+^ from B (BO) | 0.95 × 10^−3^ ms^−1^ |
| *k_*disso2 | Disassociation rate of CaMCa4 from BP | 0.7 × 10^−7^ ms^−1^ |
| *k_*dissoCa*2* | Disassociation rate of Ca^2+^ from BP | 0.95 × 10^−6^ ms^−1^ |
| *k_*cat | Transition rate from B to BP | 6.0 × 10^−5^ ms^−1^ |
| *k*cat_PP1 | Transition rate from BP to B | 1.72 × 10^−3^ ms^−1^ |
| *k_*ox | Transition rate from B to BO | 6.48 × 10^−6^ mM^−1^ ms^−1^ |
| *k_*MsrA | Transition rate from BO to B | 1.0 × 10^−4^ ms^−1^ |
